# Structural Evolution of the ABC Transporter Subfamily B

**Published:** 2007-11-08

**Authors:** J.U. Flanagan, T. Huber

**Affiliations:** 1 ARC Special Research Centre for Functional and Applied Genomics, Level 5, Institute for Molecular Bioscience, The University of Queensland, St Lucia, QLD 4072, Australia.; 2 School of Molecular and Microbial Science, The University of Queensland, St Lucia, QLD 4072, Australia.

**Keywords:** comparative modelling, ABC transporter, structure, electrostatic potential

## Abstract

The ATP binding cassette containing transporters are a superfamily of integral membrane proteins that translocate a wide range of substrates. The subfamily B members include the biologically important multidrug resistant (MDR) protein and the transporter associated with antigen processing (TAP) complex. Substrates translocated by this subfamily include drugs, lipids, peptides and iron. We have constructed a comprehensive set of comparative models for the transporters from eukaryotes and used these to study the effects of sequence divergence on the substrate translocation pathway. Notably, there is very little structural divergence between the bacterial template structure and the more distantly related eukaryotic proteins illustrating a need to conserve transporter structure. By contrast different properties have been adopted for the translocation pathway depending on the substrate type. A greater level of divergence in electrostatic properties is seen with transporters that have a broad substrate range both within and between species, while a high level of conservation is observed when the substrate range is narrow. This study represents the first effort towards understanding effect of evolution on subfamily B ABC transporters in the context of protein structure and biophysical properties.

## Introduction

The ATP-binding cassette (ABC) superfamily of transporters translocate a wide range of compounds across cellular membranes using the energy released through ATP hydrolysis. They are integral membrane proteins that have an all helical transmembrane domain (TMD) consisting of between 6 and 11 helices and an intracellular domain containing the ATP-binding cassette (nucleotide binding domain, NBD). The NBD has a Walker A and Walker B motif separated by the ABC signature, LSGGQ. These proteins have a common domain arrangement of two TMDs and two NBDs and are either produced as a single polypeptide (full transporter) or as half transporters containing one NBD and one TMD (for reviews see (Davidson and Chen, 2004; Dean and Annilo, 2005)).

Multiple ABC transporter genes exist in the genomes of bacteria and eukaryotes (Saurin et al. 1999) and, in humans, there are 48 genes divided into 8 subfamilies based on sequence analysis of the NBDs (Dean and Annilo, 2005). Loss of function mutations are associated with complex diseases that include Cystic Fibrosis, various retinal degeneration disorders and a number of liver conditions (Dean, 2005; Klein et al. 1999), while transporter overexpression and increased efflux activity is associated with the development of drug-resistance in cancers (Chen et al. 1986; McDevitt and Callaghan, 2007; Riordan and Ling, 1985; Roninson et al. 1986). Increased transporter activity may also be involved in the development of a multi-drug resistance phenotype in bacteria and other pathogenic organisms (van Veen et al. 1998; van Veen et al. 1996).

The ABC subfamily B consists of full and half transporters that have a diverse range of biological roles. Members of the subfamily include MDR1, initially identified in drug-resistant cancer cell lines (Chen et al. 1986; Juliano and Ling, 1976), and TAP, the heterodimeric peptide transporter involved in the formation of the major histocompatibility complex. Interestingly, mutations in the TAP1 and TAP2 genes, although rare (de la Salle et al. 1999), give rise to immunodeficiency disorders. Other members have roles in translocation of iron, lipids and bile salts.

Recently, the first complete and functionally relevant structure of a homodimeric subfamily B member, Sav1866, from the bacterium *S. aureus*, was reported (Dawson and Locher, 2006). Within the NBD, the Walker A and B, P-loop and ABC signature motifs overlay closely with NBDs of other bacterial transporters indicating structural conservation around the ATP binding site. Significantly, this structure illustrates the presence of a cavity at the interface of the two transmembrane subunits that is closed at the NBD end, and likely to be located in the cytoplasm, while an open conformation is adopted at the opposite end, proposed to be in the extracellular space. Residues associated with substrate binding in MDR map to this cavity further supporting its potential as the translocation pathway (Loo and Clarke, 2001; Loo and Clarke, 2002; Maki et al. 2006). Similar cavities are observed in crystal structures of other membrane transporters (Dawson and Locher, 2006; Pinkett et al. 2007). To maximise the effect of this structure in understanding the evolution of function within the ABC subfamily B, we have clustered subfamily members based on comparison of full length sequences, and used the alignment to comparatively model representative members. These models provide a structural context within which sequence divergence and effects on substrate specificities of this subfamily can be interpreted.

## Methods

### Sequence identification and selection

A total of 116 amino acid sequences including the eukaryotic ABC transporter subfamily B and bacterial orthologs of the Sav1886 protein from *S. aureus* were used in this analysis. Eukaryotic sequences were downloaded from the National Center for Biotechnology Information’s Homologene database (www.ncbi.nlm.nih.gov/entrez/query.fcgi?db=homologene). Clusters of homologous protein sequences for all 12 members of the ABC subfamily B were obtained (Homologene cluster identifiers are: tap1(B2), 495; tap2(B3), 37323; B1a, 55496; B1b, 69084; B4, 56421; B5, 83488; B6, 83488; B7, 3175; B8, 5203; B9, 10491; B10, 6474; B11, 74509.

Bacterial orthologs of Sav1886 were identified in the IMG database (http://img.jgi.doe.gov/) (Markowitz et al. 2006) of pre-computed clusters of best pair-wise BLAST alignments (ortholog cluster 7683, 37 sequences). The sequence of Sav1866 was included in the bacterial set as a reference.

### Sequence alignment, clustering and model generation and analysis

Sequence alignment and structural comparison of gene products within the subfamily B of ABC transporters is a complex problem that has necessitated some approximations. The most significant of these, is the alignment of single polypeptides that form a functional full transporter against single polypeptides that form half transporter units that are only functional as dimers. To address this, the half transporter sequences were duplicated to represent a “pseudo-full-transporter” which allowed comprehensive alignment over the full transporter sequences. As there is substantial sequence difference between eukaryotic and bacterial ABC transporter sequences, the multiple sequence alignment was constructed in two phases. Initially, separate multiple sequence alignments were computed for eukaryotes and bacteria, 102 and 14 sequence sets respectively. The alignments were then combined using a profile approach to avoid misalignment resulting from the construction of pseudo-full-transporter sequence. Due to its efficiency with this sized data set, the guide tree directed neighbour joining method as implemented in CLUSTALW (Thompson et al. 1994) was employed, followed by the profiles method. The profile alignment method relies more on conservation of amino acid properties rather than the specific residue and is well suited to the alignment of regions with remote homology, such as the transmembrane helices. In the absence of experimentally derived structural information to validate the resulting multiple sequence alignment, conservation of functional residues that occupy functionally equivalent positions in the model structures, such as the well-known Walker A and B motifs in the NBD, were used for quality assessment of the alignment. Conservation of these regions was observed between each NBD of the full transporters and the half transporter units represented as pseudo-full-transporters. As the transmembrane domains have no equivalent anchor points, the alignment through this region was assessed by superposition of gap sites on the template structure. It was clear that all gaps were located outside the helical regions in the template structure, illustrating that the conservation of residue properties across the transmembrane domain between the eukarotic and prokaryotic sequences in this study is strong even though sequence identity is low. Sequences were clustered using the neighbour joining method of CLUSTALW with 100 bootstrapped replications, and the results visualised using TREEVIEW (Page, 1996) and used to aid the interpretation of biophysical properties.

Atomic models were computed using MOD-ELLER8.2 (Fiser and Sali, 2003) with default settings. The structure of Sav1866 (PDB 2HYD) was used as the template, and pairwise alignments between the template and target sequences were extracted from the multiple sequence alignment. Half transporters were modelled as homodimers in analogy to the template structure, with exception of the TAP complex which was modelled as a heterodimer of the B2 and B3 gene products. Full transporters were modelled in the absence of the interconnecting loop between the two half transporter units. A ten model ensemble was computed for each transporter and the best scoring model, determined by the lowest MODELLER objective function value, was used for further analysis.

Electrostatic surfaces were generated by solving the non-linear Poisson-Boltzmann equation using the APBS package (Baker et al. 2001) and visualised within PyMol (DeLano, 2002). APBS was used with default settings and boundary conditions except the charge disc and surface calculation method options of multiple DH spheres, cubic b-spline and cubic spline respectively were chosen. APBS PQR input files were generated using the PDB2PQR web service (Dolinsky et al. 2004). The solvent accessible surface was coloured with a temperature spectrum describing electrostatic potential using a range of −10 to +10 kT.

All data including sequences, multiple sequence alignment and model coordinates for all ABC transporters in this study are freely available from our web site http://foo.maths.uq.edu.au/~huber/ABC/abcB.html.

## Results and Discussion

### Clustering of gene products of the ABC subfamily B

**Figure 1.**
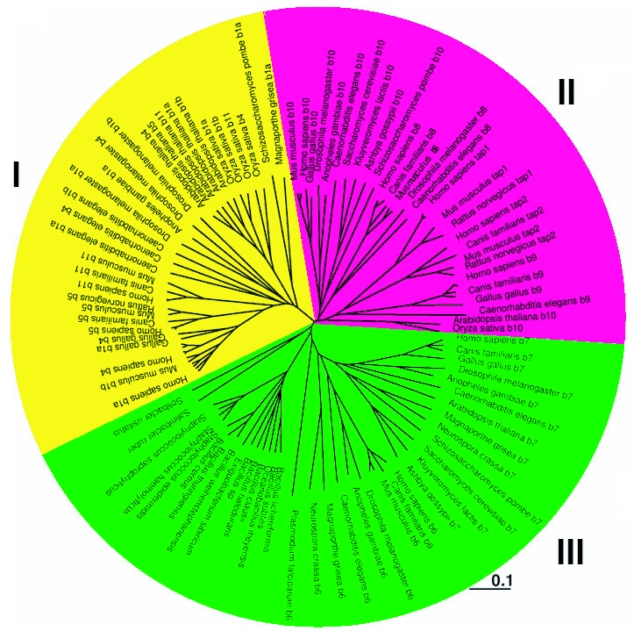
Unrooted phylogenetic tree of transporter proteins in the ABC B subfamily. Biological processes have been associated with lineages based on the clustering of proteins with characterised proteins within them. The biological functions represented include drug/lipid/bile salt (cluster I, yellow), peptide (cluster II, magenta) and iron (cluster III, green) transport. For clarity, overlapping labels from close homologous sequences were omitted.

Evolutionary analysis of ABC transporters is an extremely challenging problem, with factors such as different architectures and differing degrees of sequence divergence between domains, a likely necessity for development of biological function, requiring consideration. It is further complicated by integration into contexts of protein structure and biochemical function. Table 1 lists the ABC subfamily B genes used in this study along with their architecture, while Table 2 lists the alias, architecture and substrate type associated with the human transporter gene products. Figure 1 shows an unrooted tree that illustrates clustering of the 116 subfamily B sequences from eukaryotes and bacteria used in this study. The subfamily partitions into three main clusters, each with distinct substrate specificities. Cluster I contains the B1, B4 and B11 gene products, characterised biochemically as drug, lipid and bile salt transporters. These are primarily of full transporter architecture, with the exception of the half transporter B5, also involved in drug transport. Cluster II is composed of the half transporters B2 (TAP1), B3 (TAP2), B8, B9 and B10, and are responsible for peptide translocation across intracellular and mitochondrial membranes. These isoforms appear to have evolved according to their subcellular localization, in that B8 and B10 are located in the inner mitochondrial membrane and are clearly separated from the cellular transporters B2, B3 and B9, located in ER and lysosomal compartments, respectively. The half transporters B6 and B7 form cluster III, and are associated with iron transport. Of these three clusters, the last group is most closely related to the *S. aureus* transporter, Sav1866, for which the full structure was recently determined. Overall, the higher eukaryotes used in this study have a larger number of full transporters, likely the result of gene duplication and fusion that, along with divergence in amino acid sequence has allowed diversification in functions and substrates of this gene family.

The sequence identity between Sav1866 and members of the eukaryotic ABC subfamily B is in the range 25 to 35% which constitutes a remote sequence relationship but allows template-based comparative modelling of these transporters. Structural models have been constructed for the products of all eukaryotic genes in this study (Table 1). The half transporters B9 (Wolters et al. 2005), B10 (Galluhn and Langer, 2004; Graf et al. 2004), B6 (Krishnamurthy et al. 2006), B8 (Hogue et al. 1999) and B7 (Chloupkova et al. 2004; Csere et al. 1998) were modelled as homodimers based on experimental observations, while the TAP complex was modelled as a heterodimer as reported by Kelly et al. (Kelly et al. 1992) with B2 and B3 assuming symmetric positions in the complex. Although the quaternary state of B5 is unknown, it has been modelled conservatively as a homodimer as it is the only half transporter associated with drug translocation. Analysis of the alignment between the 11 human transporters and the distantly related bacterial template clearly illustrates that although there is much sequence divergence, the location of structural variability as indicated by insertions and deletions is outside the core of the protein.

Insertions and deletions were observed both in the extracellular and intracellular loops of the TMD and some minor length variations were also seen in the surface regions of the NDB. This indicates that the functional regions of the protein, the translocation pathway and the ATP binding site impose strong structural constraints on the evolution of these proteins. This may suggest that amino acid type distribution in the translocation pathway and resulting surface properties are likely to influence the distinct subfamily B substrate selectivity.

## Electrostatics of Translocation Pathway

**Figure 2.**
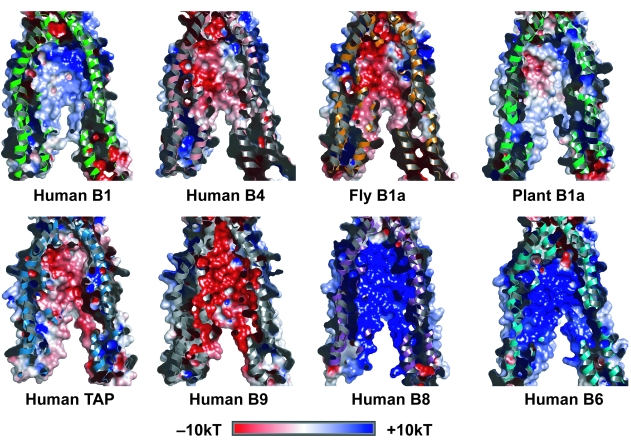
Comparative summary of electrostatic surfaces for the translocation pathway of representative transporters from human juxtaposed with examples from fly (*D. melanogaster*) and plant (*A. thaliana*). The panels are cut away views illustrating part of the surface of the translocation pathway formed by the interface of two half transporter units. Electrostatic potentials from −10 to +10 kT are displayed on the surface; red represents areas of negative electrostatic potential and blue areas represent positive electrostatic potential.

To understand more the functional influence of evolution on translocation pathways of subfamily B transporters in higher eukaryotes, and its relationship to substrate specificity, electrostatic potential was computed for a range of related sequences from different organisms, mapped to the solvent accessible surface of the translocation pathway, and interpreted within the taxonomic lineage described by the NCBI Taxamonic database (http://www.ncbi.nlm.nih.gov/Taxonomy). Figure 2 is a comparative summary of these surfaces for the translocation pathway of representative human gene products.

Human B1, well-characterised as a drug transporter, has a concentrated strong positive electrostatic potential, >10kT toward the substrate entrance of the translocation pathway. This positive charge decays along the pathway, giving a more neutral electrostatic character closer to the substrate exit site. The bile salt transporter B11 has similar surface properties (not shown). In comparison, B5, also associated with drug transport, has a positive electrostatic potential of >8kT covering the full length of the translocation pathway (not shown). In contrast to the drug transporters, B4, which has phosphatidyl choline as a substrate, has a concentrated negative electrostatic potential (<–8kT) that decays to neutral toward the substrate exit. The mouse and rat transporters display similar electrostatic properties to human B1, B4, and B5. Notably, the rodent B5 gene product unlike its human homolog, has a predominantly strong negative surface throughout the pathway, and a concentrated patch of positive potential around the substrate entrance site. This particular pathway electrostatic surface is specific to rodents. The more divergent *D. melanogaster* transporters have adopted a predominantly negative electrostatic potential similar to human B4. Clearly, during the long evolutionary period of separation between Protostomia and Deuterostomia that has lead to the insect and vertebrates included in this study, these transporters have evolved different electrostatic properties which are likely to reflect differences in the substrate(s) transported. Substrate specialisation within the subfamily is also supported by a weak similarity in charge properties observed between the plant B1 a protein from *A. thaliana* and human B1 gene product.

The human TAP peptide transporter complex has a strip of concentrated negative surface potential that extends the length of the translocation pathway and decays towards neutral over the remainder of the surface. The rat complex is similar in that it has strong negative potential throughout the pathway (not shown), while the mouse TAP translocation pathway surface differs in that it has concentrated patches of strong positive potential at both ends, decaying to a centrally located band of neutral to weak negative electrostatic potential (not shown). The lysosomal peptide transporter B9, which is more closely related to the TAP gene products rather than the mitochondrial peptide transporters, also possesses a strong negative surface potential throughout the translocation pathway, a property also conserved in the rodent B9 gene product (not shown). In contrast, the inner mitochondrial membrane peptide transporters, B8 and B10, have strong positive electrostatic potential throughout the pathway, which is also conserved in related proteins from fly (*D. melanogaster*) and plant (*A. thaliana*) (not shown). This is consistent with their clustering as distant relatives to the B9 and TAP gene products (Fig. 1). Taken together, these results illustrate that, within this cluster of peptide transporters, the translocation pathway electrostatic surface properties partition with subcellular location into two smaller clusters consisting of B8 and B10, and B2, B3 and B9. This implies that mitochondrial peptides require a pathway with a positive electrostatic surface, while those in other cellular compartments need a more negative surface, a property that is conserved across species.

The human iron transporters, B6 and B7, both have electrostatic potential surfaces similar to the human drug transporter B5 in that they have a positive charge throughout the translocation pathway. This is clearly important in the biological function of iron transport as it has also been conserved in the gene products from *D. melanogaster* and *A. thaliana*.

## Conclusion

We have developed a comprehensive set of structural models for eukaryotic members of the ABC transporter subfamily B, and used these to better understand the effects of evolution on electrostatic properties of the translocation pathway within the subfamily. Comparison of these models indicates there are no substantial structural changes in the transporter core, implying evolutionary pressure to maintain a pathway through the lipid bilayer for the ATP dependent translocation of substrates. As the surface of the translocation pathway is important for substrate selection and movement, we analysed the electrostatic properties of a number of subfamily B representatives, from different species, covering the three clusters into which this subfamily partitions. In general, each cluster is associated with a distinct substrate class, which is reflected in the different electrostatic potential properties adopted by the pathway surface. Furthermore, there is divergence of electrostatic properties within the clusters between members within a species and between species likely reflecting the range or origin of substrates recognised and translocated. This divergence is most clearly seen in the cluster of transporters that translocate diverse substrates such as drugs, lipids, bile salts, and peptides, while the more restricted substrate range of the iron transporters is reflected in the conservation of their electrostatic properties. Interestingly, the electrostatic surface for human B1 reflects the physicochemical properties of substrates for this transporter, which are lipophilic compounds possessing a tertiary basic amine, giving a positive centre, while the negative surface of the phosphotidyl choline transporter B4 reflects the negative charge associated with the phosphate and acidic groups on its phospholipid substrate. This apparent mirroring of electrostatic properties is also observed in the iron transporters, in that they have a positive electrostatic surface and transport a positively charged substrate, this similarity is unexpected as is counterintuitive, but may indicate that iron is transported in complex. Furthermore, it is possible that these properties change along with conformational rearrangements associated with ATP binding.

## Figures and Tables

**Table 1.** Distribution of ABC subfamily B members based on classification by Homologene.

**Organism**	**Subfamily B member**											
	**1A**	**1B**	**2**	**3**	**4**	**5**	**6**	**7**	**8**	**9**	**10**	**11**
Homo sapiens	F	-	H	H	F	H	H	H	H	H	H	F
Pan troglodytes	F	-	H	-	F	H	H	-	H	H	-	F
Canis familiaris	F		-	H	-	H	H	H	H	H	-	F
Mus musculus	F	F	H	H	F	H	H	H	H	H	H	F
Rattus norvegicus	F	F	H	H	F	H	H	H	H	H	H	-
Gallus gallus	F	-	-	-	F	-	H	H	-	H	H	-
Drosophila melanogaster	F	F	-	-	F	-	H	H	H	-	H	-
Caenorhabditis elegans	F	F	-	-	F	-	H	H	H	H	H	-
Oryza sativa (japonica cultivar-group)	F	F	-	-	F	-	-	-	-	-	H	F
Arabidopsis thaliana	F	F	-	-	F	H	-	H	-	-	H	F
Anopheles gambiae	F	-	-	-	-	-	H	H	-	-	H	-
Magnaporthe grisea 70–15	F	-	-	-	-	-	H	H	-	-	-	-
Schizosaccharomyces pombe 972h	F	-	-	-	-	-	H	H	-	-	H	-
Saccharomyces cerevisiae	-	-	-	-	-	-	-	H	-	-	H	-
Kluyveromyces lactis	-	-	-	-	-	-	-	H	-	-	H	-
Ashbya gossypii ATCC10895	-	-	-	-	-	-	-	H	-	-	H	-
Plasmodium falciparum 3D7	-	-	-	-	-	-	H	-	-	-	-	-
Neurospora crassa OR74A	-	-	-	-	-	-	H	H	-	-	-	-

**Abbreviations:** F: Full transporter; H: Half transporter; -: no homolog.

**Table 2.** Classification of human ABC subfamily B transporters, including alias, transporter architecture and substrate specificities.

**Gene**	**Alias**	**Architecture**	**Substrate**
ABCB1	PGY1, MDR	F	Drug
ABCB2	TAP1	H	Peptide (ER)
ABCB3	TAP2	H	Peptide (ER)
ABCB4	PGY3	F	PC
ABCB5		H	Drug
ABCB6	MTABC3	H	Fe (OM)
ABCB7	ABC7	H	Fe/S cluster (IM)
ABCB8	M-ABC1	H	Peptide (IM)
ABCB9	TAPL	H	Peptide (LY)
ABCB10	M-ABC2	H	Peptide (IM)
ABCB11	SPGP	F	Bile salt

**Abbreviations:** F: Full transporter; H: Half transporter; OM: outer mitochondrial membrane; IM: inner mitochondrial membrane; LY: lysosome.

## Abbreviations:

*A. thaliana*: *Arabidopsis thaliana;*
*D. melanogaster*: *Drosophilia melanogaster;*
*S. aureus*: *Staphylococcus aureus;*
ABC: ATP binding cassette;
TAP: Transporter associated with antigen processing.
